# Microbiological and Nutritional Quality of the Goat Meat by-Product “Sarapatel”

**DOI:** 10.3390/molecules19011047

**Published:** 2014-01-16

**Authors:** Luciana Brasil, Angela Queiroz, Josevan Silva, Taliana Bezerra, Narciza Arcanjo, Marciane Magnani, Evandro Souza, Marta Madruga

**Affiliations:** Post-Graduate Program in Food Science and Technology, Technology Centre, Federal University of Paraiba (UFPB), João Pessoa CEP 58051-900, PB, Brazil; E-Mails: luciana-vet@uol.com.br (L.B.); angelalimas@gmail.com (A.Q.); josevan.ufpb@yahoo.com.br (J.S.); taliana.kenia@hotmail.com (T.B.); narciza_moa@hotmail.com (N.A.); magnani2@gmail.com (M.M.); evandroleitesouza@hotmail.com (E.S.)

**Keywords:** non-carcass components, goat products, microbiological safety, viscera

## Abstract

Goat “sarapatel” is a product made from blood and viscera. For the first time, the microbiological and nutritional quality of “sarapatel” samples (*n* = 48) sold under different conditions (in street markets, butcher shops, and supermarkets under refrigeration, frozen or at room temperature) was evaluated. Goat “sarapatel” is a nutritive food, with each 100 g providing, on average, 72 g of moisture, 2 g of ash, 18 g of protein, 9 g of lipids, 2 g of carbohydrates, 282 mg of cholesterol, and high amounts of unsaturated fatty acids and essential amino acids. The analysis of the “sarapatel” samples shows that none of them contain *Salmonella* spp. or *L. monocytogenes*. High counts (>104) of total coliforms, thermotolerant coliforms, and sulfite-reducing *Clostridium* were detected, and coagulase-positive *Staphylococcus* was found in 31.25% of samples. The storage conditions evaluated (refrigeration, frozen or at room temperature) did not affect the physicochemical quality of the “sarapatel”; however, the unsatisfactory microbiological quality indicates that it is necessary to improve the health-sanitary aspects of the processing and sale of this product.

## 1. Introduction

Goat slaughter by-products are divided into two categories, edible and inedible. Edible by-products, such as blood and viscera, are intended for direct human consumption (fresh and semi-processed) or for the manufacture of meat products, whereas inedible products are used in the preparation of animal feed, cosmetics, and pharmaceuticals [1,2]. In the meat industry, a major goal is to convert as much of the marketable slaughter by-products as possible to reduce the environmental impact and improve economic performance or, at least, reduce the cost of waste management [2,3].

In Brazil, sheep and goat viscera and blood are frequently used in the preparation of regional dishes such as “buchada,” which is chopped meat, and “sarapatel,” in which the heart, lung, liver, intestines, and stomach are the main organs used in the preparation.

Goat “sarapatel” is a dish prepared with edible non-carcass components, including organs, viscera, and blood. This typical recipe of the Brazilian northeast is widely consumed and recognized as an important economic means of adding value to the by-products generated from goat slaughter. However, the formulation of this product is not standardized, which can lead to wide variation in composition and, consequently, nutritional quality. Goat viscera and blood have protein and lipid contents similar to those found in goat meat and are excellent sources of minerals, particularly iron, phosphorus, and vitamins [4,5]. However, the use of goat slaughter by-products in dishes such as goat “sarapatel” should adhere to the health-sanitary standards required by food laws, which would help ensure the microbiological stability of the product, increase its shelf life and maintain its physical, chemical and nutritional characteristics during storage [6].

Few studies have focused on the evaluation of the health and nutrition quality aspects of processed meat products made with goat blood and viscera, yet such products are often cited as viable alternatives for marketing and for income generation in the goat production chain. Previous studies conducted by our research group have found a high potential for the reuse of goat slaughter by-products in foods such as paté [7], goat “buchada” [8], and smoked goat sausage [9], among others. However, to the best of our knowledge, there are no scientific reports on the assessment of the health-sanitary quality and nutritional value of goat “sarapatel.” In this context, the present study aimed to evaluate the microbiological quality and some physicochemical and nutritional properties of goat “sarapatel” sold in different shops and stored under different conditions.

## 2. Results and Discussion

### 2.1. Microbiological Quality Evaluation

The microbiological analyses results revealed an absence of *Salmonella* spp. and *L. monocytogenes* in all goat “sarapatel” samples evaluated. However, 15 samples (31.25%) showed counts of coagulase-positive *Staphylococcus* above the limits (5 × 10^2^ CFU/g) established by the Brazilian legislation (Resolution RDC No. 12, item (i), with respect to blood-based products and processed derivatives) [10]. High counts of total coliforms (1.5 × 10^4^ to > 1.1 × 10^5^ MPN/g), thermotolerant coliforms (2.4 × 10^4^ to > 1.1 × 10^5^ MPN/g) and sulfite-reducing *Clostridium* (3.2 × 10³ to > 9.8 × 10^5^ MPN/g) were found in all samples (Table 1). These data reveal unsatisfactory microbiological quality of the evaluated goat “sarapatel” samples. Therefore, better sanitary conditions throughout the processing, storage, and marketing of the product should be required to improve its microbiological quality and reduce the risks to consumers.

Queiroz *et al.* [8] and Costa *et al.* [6] assessed the microbiological quality of goat “buchada” (another product made from goat slaughter by-products) and reported high counts of total and thermotolerant coliforms, which ranged from 2.4 × 10^4^ to 7.5 × 10^6^ MPN/g and from 2.4 × 10^5^ to 1.1 × 10^5^ MPN/g, respectively. Based on the counts detected in the microbiological analyses, these authors suggested that the product is outside of the microbiological standards established by legislation and is subjected to inadequate health-sanitary conditions during processing. Because the microbiological counts found in this study were similar to those detected by Queiroz *et al.* [8] and Costa *et al.* [6], it could be concluded that the analyzed goat “sarapatel” samples were processed under unsatisfactory sanitary conditions.

The presence of coagulase-positive *Staphylococcus* was detected in samples purchased in local supermarkets, both in samples kept under refrigeration and in those kept frozen. The counts of coagulase-positive *Staphylococcus* ranged from 4.3 × 10^4^ to 1.2 × 10^5^ CFU/g, and according to Aydin *et al.* [11], the presence of coagulase-positive *Staphylococcus* is related to food contamination by manipulators and inadequate cleaning and sanitization of the surfaces, utensils, materials, and equipment used during the production process.

All samples showed high counts of sulfite-reducing *Clostridium* (3.2 × 10 ³ up to > 9.8 × 10^5^ MPN/g), which pose a potential risk to public health. The formation of spores by these bacteria allows survival under processing and storage conditions, if other extrinsic conditions (pH, Aw and acidity) are conducive. Goat “sarapatel” samples showed high Aw (0.97 and 0.99), a pH close to neutral (4.68 to 6.43), and low acidity (0.49 to 0.86), conditions which favor bacterial contamination [12]. Drosinos *et al.* [13] and Guerrero-Legarreta [12] reported that the initial microbial load of by-products is related to contamination during slaughter and evisceration and to poor storage, marketing and transportation conditions.

### 2.2. Nutritional Quality Evaluation

Goat “sarapatel” samples sold under different conditions showed significant differences (*p* < 0.05) in their chemical composition parameters (Table 2). These results can be justified by the fact that goat “sarapatel” is a handmade product, with variations in the percentage of blood, viscera, and spices used in the preparation. On average, every 100 g of goat “sarapatel” contained 72 g of moisture, 2 g of ash, 18 g of proteins, 9 g of lipids and 2 g of carbohydrates.

The proximate composition of the evaluated goat “sarapatel” samples is in accordance with the literature, which shows that edible animal by-products should be recognized as significant sources of nutrients, particularly protein, and thus, these products provide an interesting opportunity to increase the nutritional quality of food products [14,15].

**Table 1:** Microbiological quality of goat “sarapatel” samples marketed under different storage conditions (results expressed as minimum and maximum value for each storage condition).

Parameters	Marketing conditions ^1^						Limit *
	PS1 (± 30 °C)	PS2 (± 10 °C)	PS3 (± −7 °C)	PS4 (± 10 °C)	PS5 (± −7 °C)	PS6 (± 10 °C)	
Total coliforms (MPN/g)	2.4 × 10^4^ to > 1.1 × 10^5^	4.6 ×10^4^ to > 1.1 × 10^5^	4.3 × 10^4^ to > 1.1 × 10^5^	1.5 × 10^4^ to > 1.1 × 10^5^	2.4 × 10^4^ to > 1.1 × 10^5^	> 1.1 × 10^5^	-
Thermotolerant coliforms (MPN/g)	9.3 × 10^4^ to > 1.1 × 10^5^	9.3 × 10^4^ to > 1.1 × 10^5^	2.9 × 10^4^ to > 1.1 × 10^5^	2.5 × 10^4^ to > 1.1 × 10^5^	2.4 × 10^4^ to > 1.1 × 10^5^	4.3 × 10^4^ to > 1.1 × 10^5^	5 × 10³
Sulphite-reducing *Clostridium* (CFU/g)	4.3 × 10^4^ to 3.0 × 10^5^	5.8 × 10^3^ to 9.8 × 10^5^	1.3 × 10^4^ to 1.0 × 10^4^	5.1 × 10^3^ to 7.0 × 10³	3.6 × 10^3^ to 1.0 × 10^5^	3.2 × 10³ to 8.0 × 10^4^	3 × 10³
Positive-coagulase *Staphylococcus* (CFU/g)	Absent	Absent	Absent	Absent	4.3 × 10^4^ to 4.4 × 10^4^	9.2 × 10^4^ to 1.2 × 10^5^	5 × 10^2^

^1^ PS1—Street market exposed for sale at temperature ±30 °C; PS2—Street market exposed for sale under refrigeration ±10 °C; PS3—butcher shop exposed for sale under freezing ± −7 °C; PS4—butcher shop exposed for sale refrigerated ±10 °C; PS5—supermarket exposed for sale under freezing ± −7 °C; PS6—supermarket exposed for sale under refrigeration ±10 °C; * BRASIL (2001)—Limit as Brazilian legislation for microbiological parameters in food.

**Table 2:** Physicochemical quality (g/100 g) of goat “sarapatel” marketed under different storage conditions.

Parameters	Marketing/storage conditions ^1^						
	PS1	PS2	PS3	PS4	PS5	PS6	General average
Moisture	72.59 ^b^ ± 1.73	67.65 ^b,c^ ± 4.55	69.27 ^c^ ± 4.65	67.27 ^c^ ± 1.63	78.34 ^a^ ± 2.08	78.81 ^a^ ± 0.97	72.54 ± 5.20
Protein	17.87 ^a,b^ ± 2.03	19.72 ^a^ ± 1.49	16.04 ^b^ ± 0.97	17.34 ^b^ ± 0.67	16.50 ^b^ ± 2.00	15.91 ^b^± 0.78	18.02 ± 1.44
Lipids	6.08 ^b^ ± 1.17	3.68 ^b^ ± 0.86	10.82 ^a^ ± 4.54	11.87 ^a^ ± 2.54	3.62 ^b^ ± 0.79	3.68 ^b^ ± 0.86	9.14 ± 3.79
Ash	1.87 ^b^ ± 0.51	3.30 ^a^ ± 0.36	2.74 ^b^ ± 0.75	2.08 ^b^ ± 0.22	0.54 ^c^ ± 0.07	0.55 ^c^ ± 0.05	1.72 ± 1.05
Carbohydrates ^2^	1.57 ^b^ ± 1.66	5.66 ^a^ ± 2.9	1.78 ^b^ ± 1.12	1.40 ^b^ ± 1.38	0.98 ^b^ ± 0.49	1.02 ^b^ ± 0.55	2.08 ± 1.78

^1^ PS1—Street market exposed for sale at temperature ± 30 °C; PS2—Street market exposed for sale under refrigeration ± 10 °C; PS3—butcher shop exposed for sale under freezing ± −7 °C; PS4—butcher shop exposed for sale refrigerated ± 10 °C; PS5—supermarket exposed for sale under freezing ± −7 °C; PS6—supermarket exposed for sale under refrigeration ± 10 ° C; ^2^ Carbohydrates expressed by difference (100 − ∑ Moisture + ash + fat + proteins); ^a,b,c^ Different letters in the same line indicate difference by the Tukey test at 5%.

### 2.3. Fatty Acid Profile

In goats, viscera have a significant content of lipid components because there is more fat deposition in the abdominal cavity than in muscle tissues [16,17]. Goat “sarapatel” contained thirty-one fatty acids, among them, seventeen saturated fatty acids (Table 3) with linear chains (C6:0 to C12:0, C14:0 and C24:0) and an average area percentage ranging from 0.02 to 0.14 and 0.04 to 22.56, respectively; eight monounsaturated fatty acids (C14:1 to C16:1, C18:1, C20:1 and C24:1) with an average area percentage from 0.10 to 29.02; and six polyunsaturated fatty acids (C18:2, C18:3, C20:4 and C22:6), with a variation of 0.12 to 3.89 in the area percentage.

Saturated fatty acids (SFA) were predominant (58%) in goat “sarapatel” samples, among which acids C18:0 (26.80 to 31.15%), C16:0 (21.88 to 23.76%) and C14:0 (2.15 to 2.70%) stood out. The high SFA concentrations found in goat “sarapatel” samples may be related to biohydrogenation in the rumen of ruminant animals, which is a natural process carried out by ruminal microorganisms that reduces the deleterious effect of lipids in these tissues by promoting the lysis of esterified lipids, with subsequent hydrogenation of free fatty acids [18,19].

Among the detected fatty acids, monounsaturated fatty acids (MUFA), such as oleic acid—C18:1 ω-9c (24.22% to 33.41%) and vaccenic acid—C18:1 n11t (2.5 to 3.8%), also stood out, with percentages ranging from 30.97 to 40.05. Goat “sarapatel” samples also showed some content (from 4.4% to 8.5%) of polyunsaturated fatty acids (PUFA), especially conjugated linoleic acids—C18:2 ω-6 (2.33 to 5.22%) and rumenic acid—C18:2 9c, 11t-CLA (0.39 to 0.48%), which are considered essential fatty acids for humans. These acids not only play a role in the constitution of tissues of the central nervous system but also act in the prevention of cardiovascular, autoimmune and inflammatory diseases, in addition to their anti-cancer activity [20]. Vaccenic acid, the main precursor of CLA-rumenic acid [21], stood out among the detected *trans* fatty acids. Information on the fatty acid profile of goat slaughter by-products is generally scarce and is nonexistent for goat “sarapatel,” but it was observed that goat “sarapatel” had a fatty acid percentage similar to those reported for goat “buchada” [8,22] and for ovine and bovine viscera [17,21].

The PUFA/SFA ratio in goat “sarapatel” (0.07 to 0.13) was similar to those reported by Santos *et al.* [16] for goat “buchada,” which is (>0.45) below the level proposed by the UK Department of Health and Safety [23]. However, this relationship is not fully adequate, as it fails to assign a hypercholesterolemic effect to all fatty acids and therefore ignores the hypocholesterolemic effects of MUFA. The hypocholesterolemic (h) fatty acids/hypercholesterolemic (H) fatty acids ratio (h:H), which is related to greater or lesser risk of cardiovascular diseases [24], ranged from 1.28 to 1.62; these values were higher than those detected for sheep meat [25]. In addition, the mean values found for the atherogenicity (AI) and thrombogenicity indexes (TI) were 0.45 and 1.41, respectively.

These values were similar to those detected for sheep meat. According to Turan *et al.* [26], the AI and TI indicate the potential for the stimulation of platelet aggregation, and thus, the smaller the AI and TI values, the larger the amount of anti-atherogenic fatty acids present in fat and, consequently, the greater the potential for preventing the onset of coronary heart diseases.

**Table 3:** Fatty acids profile (% area) of goat “sarapatel” marketed under different storage conditions.

Parameters	Marketing/storage conditions ^1^						General average
	PS1	PS2	PS3	PS4	PS5	PS6	
C6:0	0.07 ^a^ ± 0.03	0.04 ^a^ ± 0.03	0.02 ^a^ ± 0.00	0.02 ^a^ ± 0.00	0.15 ^a^ ±0.21	0.07 ^a^ ± 0.09	0.06 ± 0.05
C8:0	0.08 ^a^ ± 0.04	0.04 ^a^ ± 0.01	0.03 ^a^ ± 0.01	0.03 ^a^ ± 0.01	0.15 ^a^ ±0.19	0.15 ^a^ ± 0.16	0.08 ± 0.06
C9:0	0.06 ^a^ ± 0.02	0.04 ^a^ ± 0.01	0.03 ^a^ ± 0.01	0.03 ^a^ ± 0.01	0.11 ^a^ ± 0.13	0.11 ^a^ ± 0.11	0.06 ± 0.04
C10:0	0.07 ^a^ ± 0.03	0.08 ^a^ ± 0.04	0.11 ^a^ ± 0.01	0.10 ^a^ ± 0.00	0.10 ^a^ ± 0.01	0.09 ^a^ ± 0.01	0.09 ± 0.01
C11:0	0.03 ^a^ ± 0.01	0.01 ^a^ ± 0.01	0.01 ^a^ ± 0.01	0.01 ^a^ ± 0.01	0.03 ^a^ ± 0.05	0.03 ^a^ ± 0.05	0.02 ± 0.01
C12:0	0.13 ^a^ ± 0.03	0.15 ^a^ ± 0.03	0.14 ^a^ ± 0.06	0.10 ^a^ ± 0.01	0.20 ^a^ ± 0.06	0.18 ^a^ ± 0.02	0.14 ± 0.04
C14:0	2.41 ^a^ ±0.05	2.70 ^a^ ± 0.16	2.65 ^a^ ± 0.46	2.15 ^a^ ± 0.08	2.66 ^a^ ± 0.28	2.40 ^a^ ± 0.04	2.49 ± 0.21
C15:0	0.71 ^a,b^ ± 0.07	0.82 ^a^ ± 0.02	0.77 ^a^ ± 0.11	0.58 ^b^ ± 0.03	0.84 ^a^ ± 0.05	0.79 ^a^ ± 0.04	0.74 ± 0.10
C16:0	22.19 ^a^ ± 0.35	22.01 ^a^ ± 1.07	22.22 ^a^ ± 1.21	21.88 ^a^ ± 0.86	23.32 ^a^ ± 0.04	23.76 ^a^ ± 1.05	22.56 ± 0.97
C17:0	1.70 ^b^ ± 0.13	1.93 ^a,b^ ± 0.12	2.34 ^a^ ± 0.14	1.90 ^b^ ± 0.17	2.00 ^a,b^ ± 0.02	1.88 ^b^ ± 0.18	1.95 ± 0.21
C18:0	30.49 ^a^ ± 3.65	31.50 ^a^ ± 2.30	26.81 ^a^ ± 2.73	27.28 ^a^ ± 0.58	26.80 ^a^ ± 1.71	27.27 ^a^ ± 1.40	28.35 ± 2.08
C19:0	0.33 ^a^ ± 0.03	0.40 ^a^ ± 0.02	0.37 ^a^ ± 0.03	0.30 ^a^ ± 0.03	0.36 ^a^ ± 0.16	0.34 ^a^ ± 0.12	0.35 ± 0.03
C20:0	0.95 ^a^ ± 0.40	0.81 ^a^ ± 0.27	0.65 ^a^ ± 0.10	0.66 ^a^ ± 0.13	0.63 ^a^ ± 0.24	0.76 ^a^ ± 0.06	0.74 ± 0.12
C21:0	0.28 ^a^ ± 0.13	0.36 ^a^ ± 0.03	0.32 ^a^ ± 0.01	0.25 ^a^ ± 0.02	0.32 ^a^ ± 0.17	0.50 ^a^ ± 0.17	0.34 ± 0.09
C22:0	0.23 ^a^ ± 0.14	0.21 ^a^ ± 0.25	0.17 ^a^ ± 0.19	0.19 ^a^ ± 0.27	0.16 ^a^ ± 0.08	0.14 ^a^ ± 0.12	0.18 ± 0.03
C23:0	0.08 ^a^ ± 0.01	0.08 ^a^ ± 0.09	0.07 ^a^ ± 0.00	0.06 ^a^ ± 0.00	0.06 ^a^ ± 0.11	0.07 ^a^ ± 0.05	0.07 ± 0.06
C24:0	0.09 ^a^ ± 0.08	0.09 ^a^ ± 0.16	0.03 ^a^ ± 0.04	0.02 ^a^ ± 0.39	0.03 ^a^ ± 0.05	0.02 ^a^ ± 0.03	0.04 ± 0.04
C14:1	0.20 ^b^ ± 0.11	0.43 ^a^ ± 0.02	0.30 ^a,b^ ± 0.02	0.31 ^a,b^ ± 0.03	0.38 ^a^ ± 0.02	0.33 ^a,b^ ± 0.05	0.33 ± 0.08
C15:1	0.32 ^a,b,c^ ± 0.04	0.38 ^a^ ± 0.02	0.29 ^b,c^ ± 0.01	0.26 ^c^ ± 0.01	0.35 ^a,b^ ± 0.01	0.33 ^a,b^ ± 0.02	0.32 ± 0.04
C16:1	1.01 ^a^ ± 0.10	1.01 ^a^ ± 0.11	1.22 ^a^ ± 0.03	1.16 ^a^ ± 0.08	1.07 ^a^ ± 0.08	1.01 ^a^ ± 0.17	1.08 ± 0.13
C18:1 n9c	24.22 ^b^ ± 5.40	26.36 ^a,b^ ± 4.25	33.41 ^a,b^ ± 1.04	34.45 ^a^ ± 0.77	27.93 ^a,b^ ± 1.57	27.77 ^a,b^ ± 1.40	29.02 ± 4.74
C18:1n9-t	0.52 ^a^ ± 0.21	0.31 ^a,b^ ± 0.01	0.27 ^a,b^ ± 0.02	0.25 ^a,b^ ± 0.06	0.20 ^b^ ± 0.04	0.21 ^b^ ± 0.03	0.29 ± 0.14
C18:1 n11c	3.79 ^a^ ± 0.51	3.81 ^a^ ± 0.37	2.98 ^a,b^ ± 0.17	3.16 ^a,b^ ± 0.62	2.48 ^b^ ± 0.10	2.60 ^a,b^ ± 0.26	3.13 ± 0.65
C20:1n9	0.15 ^a^ ± 0.04	0.10 ^a^ ± 0.03	0.07 ^a^ ± 0.02	0.09 ^a^ ± 0.00	0.11 ^a^ ± 0.04	0.08 ^a^ ± 0.06	0.10 ± 0.04
C24:1n9	0.76 ^a^ ± 0.34	0.60 ^a^ ± 0.49	0.46 ^a^ ± 0.18	0.37 ^a^ ± 0.10	0.80 ^a^ ± 0.40	1.01 ^a^ ± 0.16	0.66 ± 0.38
C18:2n6c	5.22 ^a^ ± 0.62	3.50 ^a,b^ ± 1.05	2.36 ^b^ ± 0.35	2.90 ^a,b^ ± 0.09	4.61 ^a,b^ ± 0.55	4.73 ^a,b^ ± 1.06	3.89 ± 1.26
C18:2n6t	0.07 ^a^ ± 0.06	0.13 ^a^ ± 0.01	0.14 ^a^ ± 0.01	0.12 ^a^ ± 0.02	0.13 ^a^ ± 0.00	0.12 ^a^ ± 0.03	0.12 ± 0.04
C18:3n3	0.15 ^a^ ± 0.11	0.20 ^a^ ± 0.04	0.16 ^a^ ± 0.05	0.17 ^a^ ± 0.02	0.34 ^a^ ± 0.20	0.23 ^a^ ± 0.08	0.21 ± 0.12
C18:2 9c.11t-CLA	0.38 ^a^ ± 0.06	0.44 ^a^ ± 0.04	0.47 ^a^ ± 0.11	0.48 ^a^ ± 0.11	0.44 ^a^ ± 0.03	0.46 ^a^ ± 0.10	0.44 ± 0.09
C20:4n6	1.66 ^a^ ± 0.58	1.20 ^a^ ± 0.90	1.22 ^a^ ± 0.54	1.29 ^a^ ± 0.64	2.11 ^a^ ± 0.73	2.15 ^a^ ± 0.53	1.58 ± 0.48
C22:6n3	0.08 ^a^ ± 0.52	0.21 ^a^ ± 0.19	0.34 ^a^ ± 0.20	0.40 ^a^ ± 0.25	0.88 ^a^ ± 0.09	0.58 ^a^ ± 0.41	0.42 ± 0.23
SFA	59.90 ± 8.52	61.27 ± 8.68	56.74 ± 7.85	55.56 ±7.90	57.92 ± 8.00	58.56 ± 8.23	58.26 ± 8.17
MUFA	30.97 ± 8.31	33.00 ± 9.06	39.00 ± 11.57	40.05 ± 11.94	33.32 ± 9.63	33.34 ± 9.59	34.93 ± 10.01
PUFA	7.56 ± 2.03	5.68 ± 1.31	4.69 ± 0.85	5.36 ± 1.05	8.51 ± 1.72	8.27 ± 1.80	6.66 ± 1.45
MUFA/SFA	0.52 ^a^ ± 0.19	0.54 ^a^ ± 0.02	0.69 ^a^ ± 0.13	0.72 ^a^ ± 0.09	0.57 ^a^ ± 0.11	0.57 ^a^ ± 0.11	0.60 ± 0.08
PUFA/SFA	0.13 ^a^ ± 0.01	0.09 ^a^ ± 0.04	0.08 ^a^ ± 0.02	0.09 ^a^ ± 0.02	0.15 ^a^ ± 0.01	0.14 ^a^ ± 0.04	0.11 ± 0.03
h:H	1.28 ^a^ ± 0.23	1.28 ^a^ ± 0.19	1.50 ^a^ ± 0.12	1.62 ^a^ ± 0.12	1.39 ^a^ ± 0.04	1.36 ^a^ ± 0.01	1.41 ± 0.01
AI	0.45 ^a^ ± 0.09	0.43 ^a^ ± 0.06	0.476 ^a^ ± 0.04	0.43 ^a^ ± 0.02	0.46 ^a^ ± 0.02	0.45 ^a^ ± 0.01	0.45 ± 0.06
TI	1.42 ^a^ ± 0.44	1.35 ^a^ ± 0.23	1.59 ^a^ ± 0.07	1.32 ^a^ ± 0.06	1.51 ^a^ ±0.03	1.28 ^a^ ± 0.04	1.41 ± 0.06
Cholesterol (mg/100 g)	318.58 ^a^ ± 56.75	249.87 ^a^ ± 77.83	326.14 ^a^ ± 110.39	332.73 ^a^ ± 5.58	218.98 ^a^ ± 24.23	249.76 ^a^ ± 37.61	282.67 ± 44.54

^1^ PS1—Street market exposed for sale at temperature ± 30 °C; PS2—Street market exposed for sale under refrigeration ± 10 °C; PS3—butcher shop exposed for sale under freezing ± −7 °C; PS4—butcher shop exposed for sale refrigerated ± 10°C; PS5—supermarket exposed for sale under freezing ± −7 °C; PS6—supermarket exposed for sale under refrigeration ± 10 °C; ^a,b,c^ Different letters in the same line indicate difference by the Tukey test at 5%; h:H-hypocholesterolemic-h fatty acids/hypercholesterolemic—H fatty acids ratio = (C18:1 cis9 + C18:2 ω-6 + C20:4 ω-6 + C18:3 ω-3 + C22:6 ω-3)/(C14:0 + C16:0) [24]; AI—Atherogenicity index = [(C12:0 + (4 × C14:0) + C16:0)]/ω-6 + ω-3 + MUFA + C18:1 [27]; TI—thrombogenicity index = (C14 + C16 + C18)/(0.5 × (C18:1 + MUFA + ω-6) + (3 × ω-3)+(ω-3/ω-6) [27].

The cholesterol levels in the goat “sarapatel” samples ranged from 218.98 mg/100 g to 332.73 mg/100 g, which can be explained by the fact that cholesterol is synthesized in organs such as the liver and kidneys [28], which are used as raw materials for the preparation of goat “sarapatel.” The percentage of cholesterol in edible organs and viscera can reach levels up to three to five times that of the muscle portion of the same animal [29]. The amounts of cholesterol found in goat “sarapatel” samples were in accordance with the literature for viscera of ruminants [28] and meat products prepared with goat slaughter by-products [8,9,22].

### 2.4. Amino Acid Profile

Edible goat meat by-products are recognized as having high amounts of protein (approximately 20 g/100 g) and balanced amino acid profiles, similar to those of meat protein [30]. The amounts of amino acids found in goat “sarapatel,” together with the daily intake values recommended for adequate nutrition [31] and the score for each essential amino acid identified, are shown in Table 4. The amount of essential amino acids detected in goat “sarapatel” samples represented, on average, 47% of the total content of amino acids detected, making this product a source of essential amino acids, especially lysine (297 mg/100 g), histidine (286 mg/100 g), leucine (252 mg/100 g), valine (177 mg/100 g) and threonine (138 mg/100 g).

Goat “sarapatel” showed no limiting amino acids, and the chemical average scores were above 1.0 for all essential amino acids [32]. Previous studies have shown that the use of goat slaughter by-products in the preparation of meat products such as goat “buchada” [8] and smoked goat sausage [9] has been considered a feasible alternative way to obtain food products with high protein quality, and the data shown in the present study support the fact that goat “sarapatel” is another product made with viscera and blood and a good source of high-quality protein, considering the amount of essential amino acids.

## 3. Experimental

### 3.1. Materials

According to a survey performed in the city of João Pessoa, Paraíba, Brazil, goat “sarapatel” is sold in three types of shops and under different storage conditions, *i.e.*, in street markets where it is exposed to temperatures ± 30 °C (PS1); in street market where it is refrigerated at ± 10 °C (PS2); in butcher shops where it is frozen at ± −7 °C (PS3); in butcher shops where it is refrigerated at ± 10 °C (PS4); in supermarkets where it is frozen at ± −7 °C (PS5); and in supermarkets where it is refrigerated at ± 10 °C (PS6). Based on this information, goat “sarapatel” samples were collected on a weekly basis, totaling 48 samples, and microbiological and nutritional studies were performed on eight samples for each selling condition of the product (PS1 to PS6).

**Table 4:** Amino acids profile ^1^ (mg/100 g protein) of goat “sarapatel” under different marketing conditions.

Amino acids	Marketing/storage conditions ^1^						General average	Standard FAO ^2^	AA Score ^3^
	PS1	PS2	PS3	PS4	PS5	PS6			
Essential Amino acids									
Lysine	223.72	352.81	310.06	194.55	294.21	400.21	297.59	45	4.32 to 8.89
Phenylalanine + Tyrosine	254.38	319.12	312.50	125.93	328.02	386.45	287.73	38	3.31 to 10.17
Histidine	278.87	260.97	177.11	190.32	494.54	316.91	286.45	15	11.81 to 32.97
Leucine	212.38	258.55	258.46	93.39	293.44	397.28	252.25	59	1.58 to 6.73
Valine	152.37	101.80	214.45	89.74	232.04	270.55	176.82	39	2.30 to 6.94
Threonine	91.05	151.48	142.56	110.35	152.10	180.04	137.93	23	3.96 to 7.83
Isoleucine	80.81	194.90	112.38	140.04	133.15	153.28	135.76	30	2.69 to 6.47
Methionine	65.57	73.20	59.69	36.43	63.96	77.56	62.73	22	1.65 to 3.52
Nonessential Amino acids									
Glutamic acid	279.78	319.44	333.88	259.69	383.27	461.93	339.66	NA ^4^	NA
Serine	385.29	244.69	281.55	313.82	394.00	366.76	331.02	NA	NA
Aspartic acid	220.60	266.32	291.65	213.12	320.08	388.78	283.42	NA	NA
Arginine	209.37	202.32	213.81	139.42	284.45	325.78	229.19	NA	NA
Alanin	152.00	220.24	231.90	164.37	274.36	180.04	203.82	NA	NA
Glycine	146.29	62.83	199.18	149.24	100.56	225.94	147.34	NA	NA
Proline	51.76	57.25	76.46	19.32	52.01	135.89	65.45	NA	NA

^1^ PS1—Street market exposed for sale at temperature ± 30 ° C; PS2—Street market exposed for sale under refrigeration ± 10°C; PS3—butcher shop exposed for sale under freezing ± −7°C; PS4—butcher shop exposed for sale refrigerated ± 10°C; PS5—supermarket exposed for sale under freezing ± −7 ° C; PS6—supermarket exposed for sale under refrigeration ± 10 ° C; ^2^ Values reported on a dry basis; ^2^ Estimated amino acids requirement (adults). Reference standard of protein FAO/WHO/UNU (2007); ^3^ Amino acids score (mg/g protein sample)/(mg/g protein standard FAO/WHO); ^4^ Not applicable.

### 3.2. Methods

#### 3.2.1. Microbiological Quality Evaluation

Goat “sarapatel” samples were analyzed for total coliforms (MPN/g) and thermotolerant coliforms (MPN/g), coagulase-positive *Staphylococcus* (CFU/g), sulfite-reducing *Clostridium* (CFU/g) and the presence of Salmonella spp and *Listeria monocytogenes* according to methodology described by the APHA [33]. pH, method 947.05 of the AOAC [34] and water activity, method 978.18 of the AOAC [34] were determined as extrinsic factors related to the microbiological stability of the product.

#### 3.2.2. Nutritional Quality Evaluation

The goat “sarapatel” was analyzed for its proximate composition, cholesterol content, and fatty acid and amino acid profiles. The moisture, ash and protein contents were determined using the methodology described in items 950.46.41, 928.08 and 920 153, respectively, of the AOAC [34]. The carbohydrate concentration was determined by difference (100-ΣMoisture + ashes + fat + protein). The ether extract was analyzed according to procedures described by Folch, Less and Stanley [35].

The fatty acid profile of the ether extract was submitted to methylation according to the methodology described by Hartman and Lago [36], and the identification and quantification of fatty acid esters was conducted by gas chromatography [9].

To quantify the levels of total cholesterol, isocratic elution was performed on a liquid chromatography (Varian, Harbor City, CA, USA) coupled with an Inertisil C18 column (4.6 mm × 150 mm × 5 mm); chromatographic separation was performed at constant flow (1 mL/min) at 30 °C with a run time of 10 min according to the methodology described by Bragagnolo and Rodriguez-Amaya [37].

The amino acid profile was determined according to the methodology described by White, Hart and Fry [38], in which amino acids were identified in previously hydrolyzed samples using 6 N double-distilled hydrochloric acid, followed by pre-column derivatization of free amino acids with phenylisothiocyanate (PITC). The separation of phenylthiocarbamyl-amino acid derivatives (PTC-aa) was carried out by high-resolution liquid chromatography [9].

### 3.3 Statistical Analysis

The experiment was conducted with a completely randomized design. Nutritional assessment data were submitted to analysis of variance (ANOVA), and means were compared by the Tukey test with *p* ≤ 0.05 [39].

## 4. Conclusions

The evaluated goat “sarapatel” samples had high nutritional quality, especially when considering the protein content and the amino acid and fatty acid profiles, but there was wide variation in these parameters, revealing a lack of standardization in the preparation process. The results of microbiological analyses revealed inadequate health-sanitary conditions, which pose a potential risk to consumer health and indicate the need for the adoption of measures that will improve the health-sanitary conditions during the processing, storage and marketing of goat “sarapatel.” In conclusion, the development of products based on goat slaughter by-products, particularly goat “sarapatel,” is an interesting opportunity to increase the range of goat products for use in human nutrition and to reuse these usually discarded by-products.
